# Dextromethorphan: a case study on addressing abuse of a safe and effective drug

**DOI:** 10.1186/s13011-016-0067-0

**Published:** 2016-06-23

**Authors:** David C. Spangler, Catherine M. Loyd, Emily E. Skor

**Affiliations:** Consumer Healthcare Products Association, Washington, DC, USA; Growth Energy, Washington, DC, USA

**Keywords:** Drug abuse, Substance abuse, Prevention, Dextromethorphan, At-risk youth

## Abstract

**Background:**

Dextromethorphan is a safe, effective cough suppressant, available without a prescription in the United States since 1958. Due to a perceived prevalence of abuse of dextromethorphan by teens, in 2007 the Drug Enforcement Administration requested the Food and Drug Administration evaluate whether dextromethorphan should be recommended for scheduling under the Controlled Substances Act. The Food and Drug Administration held an Advisory Committee meeting in 2010 to provide a scientific and medical evaluation of dextromethorphan and its abuse potential.

**Discussion:**

To address reports of abuse, particularly by teens in the United States, the Consumer Healthcare Products Association initiated an abuse mitigation plan in 2010 with specific goals related to awareness of the behavior, perception of risk, social disapproval, and access to the products. In identifying abuse interventions, experts acknowledge that substance abuse among teens is a highly complex behavior and indicate that the best course of action is to address prevention by focusing on the factors that impact teen behavior.

**Conclusion:**

It is noteworthy that the annual prevalence of over-the-counter cough medicine abuse has sharply decreased since 2010. While a true cause-and-effect relationship cannot be assured, the Consumer Healthcare Products Association and its member companies believe that the increased awareness of the issue since the 2010 Food and Drug Administration Advisory Committee meeting, and the subsequent implementation of a well-delivered and targeted abuse mitigation plan that addressed the levers influencing teen decisions is contributing to the observed reduction in abuse. During the period of 2010–2015, reported abuse of dextromethorphan by 8^th^, 10^th^, and 12^th^ graders decreased 35 %. The authors believe this reduction supports the view of the Consumer Healthcare Products Association at the outset of the abuse mitigation plan effort and today: Controlled substance scheduling or prescription requirements would result in a reduction in the legitimate use of this medicine that has benefits that far outweigh its risks. Instead, there are more targeted, more effective, and less disruptive interventions to address dextromethorphan abuse.

## Background

Dextromethorphan is a safe, effective cough suppressant, available without a prescription in the United States since 1958. Due to a perceived prevalence of abuse of dextromethorphan by teens, the Drug Enforcement Administration requested the Food and Drug Administration evaluate whether dextromethorphan should be recommended for scheduling under the Controlled Substances Act in 2007, and the Food and Drug Administration held an Advisory Committee meeting in 2010 to provide a scientific and medical evaluation of dextromethorphan and its abuse potential.

## Main text

### Overview of dextromethorphan (DXM)

Dextromethorphan (DXM) is a safe, cough suppressant with a long history of nonprescription or over-the-counter (OTC) use in the U.S. and many other countries. DXM was approved by the U.S. Food and Drug Administration (FDA) as an OTC antitussive in 1958. In the 1970s, FDA reviewed the available safety and efficacy data on DXM, concluding that the drug was generally recognized as safe and effective [1, 2]. While there were reports of abuse, a review panel concluded “because of its low order of toxicity, it is probably the safest antitussive presently available” [1]. Millions of Americans have and continue to rely on this accessible medicine; according to market research firm IRI, DXM-containing OTC medicines comprise 85 to 90 % of all medicines containing a cough suppressant sold in the U.S., and approximately 235 million packages of DXM-containing OTC medicines were purchased in the last year.

Acute cough is very prevalent, and multiple studies have demonstrated that a majority of Americans self-medicate for cough and the common cold [3]. People’s daily routines are significantly impaired when they suffer from acute cough, as symptoms create a burden and an economic impact in terms of lost work days, school absenteeism, and reduced work productivity [4, 5]. Access to OTCs, like DXM, enables consumers to take appropriate control of their own healthcare and provides demonstrated economic value to the healthcare system [6].

DXM is a centrally acting cough suppressant. It is believed to suppress cough by altering the threshold for cough initiation through effects in the medulla oblongata [7]. While its pharmacology is not completely understood, DXM has been shown to bind to receptors implicated in the cough response, including the sigma-1 receptors and N-methyl-D-aspartate (NMDA) receptors. Although DXM is an NMDA antagonist, it differs biochemically from high-affinity antagonists such as phencyclidine and dizocilpine [8]. DXM continues to be studied widely in combination with other active pharmaceutical ingredients, including for neurological conditions such as pseudobulbar effect and Rett syndrome [9, 10].

Although structurally similar to other morphine derivatives, DXM is a non-narcotic cough suppressant since it is devoid of morphine-like effects [11]. However, at high doses DXM can exert mixed clinical psychoactive effects which play a role in its non-medical abuse, eliciting both euphoria and dysphoria, distorted visual perceptions, loss of motor coordination, dissociative sedation, nausea, and vomiting [12]. Qualitative research among substance abusers shows little recurring abuse of DXM [11, 13, 14].

In 2006, the National Institute of Drug Abuse (NIDA) funded Monitoring the Future survey showed abuse of DXM-containing OTC cough medicine was concentrated among teens and, to a lesser degree, young adults: 5.4 % of 8th, 10th, and 12th grade students reported non-medical use of OTC cough medicine in the past year [15]. For comparison, the survey reported that 22 % used marijuana and 50.7 % used alcohol in the previous year [15].

Based on a perceived risk of increasing prevalence of abuse among teens and despite the longstanding documented safety and benefit of DXM, the Drug Enforcement Administration (DEA) in 2007 requested that FDA evaluate whether DXM should be recommended for scheduling under the Controlled Substances Act [16]. Subsequently, FDA held an Advisory Committee meeting in September 2010 to provide a scientific and medical evaluation of DXM and its abuse potential. To prepare for this meeting, the Consumer Healthcare Products Association (CHPA), representing the manufacturers of OTC medicines in the U.S., reviewed the abuse of DXM. The review was based on over 50 years of marketing experience; consultation with experts; the totality of a review of pharmacology, preclinical, and clinical study data; the prevalence of reported abuse from national, government-sponsored surveys; a review of outcomes databases; and the benefits and risks of DXM to public health [12].

### Industry and prevention expert abuse mitigation plan

Though these medicines provide relief to millions of Americans annually safely and effectively, the limited reports of abuse associated with DXM must also be addressed to maintain consumers’ safety. To directly address these reports, as early as 2003 CHPA began developing educational interventions against abuse [12]. By 2010, it was not clear whether these interventions were sufficient, at which point CHPA formalized its abuse interventions through the development of an abuse mitigation plan with participation from companies in the OTC industry and leading abuse prevention experts. In identifying cough medicine abuse interventions, experts acknowledge that substance abuse among teens is a highly complex behavior and indicate that the best course of action is to address prevention by focusing on the factors that impact teen behavior. Literature points to a number of key factors leading to the prevalence of substance abuse among teenagers, including low parental awareness, low teen perception of risk, low perception of social disapproval, and ready availability [12]. Therefore, CHPA made a commitment to try to reduce initiation of the behavior by delivering against the following goals, initially set out at the 2010 FDA Advisory Committee meeting, over a 3-year period:

- increase teen perception of risk by highlighting the physical risks of abuse;
- increase teen perception of social disapproval by emphasizing peer’s disapproval of abuse behavior and demonstrating that non-abuse is the norm;
- raise awareness of DXM abuse and risks among parents, caregivers, and teen-influencers; and
- limit teen access to DXM by increasing parental safeguarding and monitoring of DXM-containing medicines in the home and by advocating for legislation to establish a sales age restriction and prohibit the sale of bulk, unfinished DXM to parties not registered with FDA.

CHPA tracks the attitudes and behaviors of teens and parents annually via sponsored qualitative and quantitative research and available national survey data. In addition, CHPA relies on the expertise and guidance of those with demonstrated effectiveness in reducing drug abuse, such as the Partnership for Drug-Free Kids (the Partnership) [17], to develop plans that will best distribute the messages.

For example, a key strategy advocated by the Partnership and implemented in components of the CHPA program was to specifically target those teens most at risk for abuse behavior. This was feasible using an Internet-based outreach plan since data showed that teens actively go online to search for information on how to abuse DXM and to discuss the experience via social media. As OTC cough medicine abuse is not widespread among the teen population, this targeted approach also reduced the potential unintended consequence of increasing interest among non-abusers.

Thus, a portfolio of “bait-and-switch” YouTube videos, Facebook and mobile app experiences, teen testimonial videos, and a content-rich website was created in a teen “voice.” All creative elements and messages were tested with our target teen audiences prior to launch to ensure maximum impact and to reduce potential unintended consequences. The authenticity of these messages is intended to discourage teens from abusing DXM without turning them off from listening. Starting in 2012, this content was selectively seeded across the Internet where there was a high probability that potential teen abusers would be exposed.

Regarding program components that target parents, caregivers, and teen-influencers, CHPA has been educating on the issue since 2003. Following the trend toward online engagement, the Stop Medicine Abuse campaign now connects with parents and caregivers via online communities in addition to local groups familiar to parents including the Community Anti-Drug Coalitions of America, the National School Nurses Association, and other trusted sources. Contrary to the relatively small intended teen target audience, this target audience is the general parent and caregiver populations. The program’s goal is to spread awareness of the potential for abuse and encourage parents and caregivers to take the steps necessary to prevent it. The conversation between a parent and their child is critical, as teens are up to 50 % less likely to use drugs if they learn about the risks from their parents [18].

**Fig. 1:**
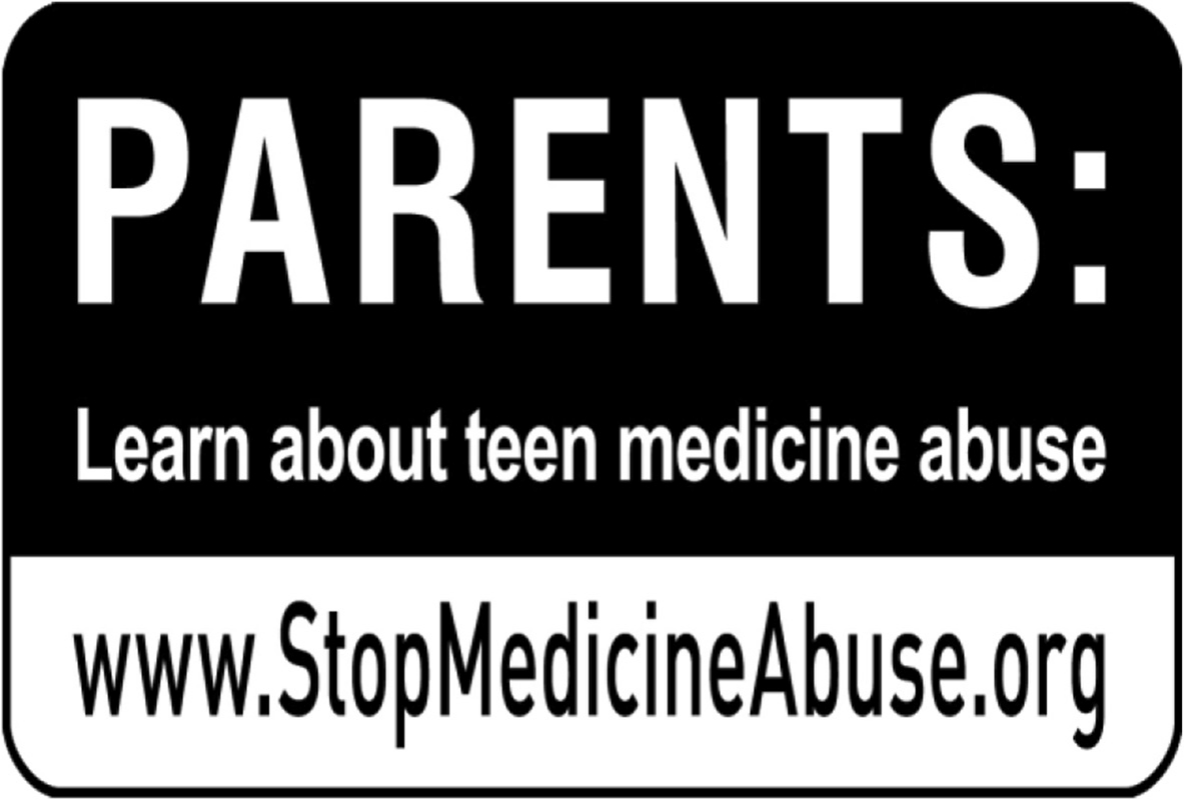
Icon placed on most packages containing DXM

In addition, beginning in 2008, with expansion through 2010, CHPA member companies placed an icon on packages of DXM sold in the U.S. alerting parents and caregivers to the issue and directing them to StopMedicineAbuse.org. There, they can learn about DXM abuse, its side effects and warning signs, and tools on how to prevent this behavior. CHPA estimates the icon is placed on more than 235 million packages each year (Fig. 1).

Finally, access to DXM medicines can be limited by parents in the home, and the CHPA campaign continues to actively promote the safe storage and monitoring of DXM-containing medicines. Further, the CHPA program supports national and state legislation to limit minors’ access to the medicines by requiring that consumers be at least 18 years old to purchase DXM, and by prohibiting the sale of bulk, unfinished DXM to parties not registered with FDA. With limited access, the prevalence rate should decrease, as seen in youth tobacco use. The campaign to reduce youth smoking has included many tactics, but the correlation between youth smoking and the sales restrictions to minors stands out. Since the 1992 Synar Act, which required states to prohibit sales or distribution of tobacco to minors, was implemented in 1996 by the Substance Abuse and Mental Health Service Administration (SAMSHA) [19], the levels of past 30-day smoking have fallen by about 80 % among 8^th^ and 10^th^ graders and by more than 65 % among 12^th^ graders [20]. Therefore, CHPA is active in both direct lobbying and providing portals for opinions of constituents to their representatives in Congress or state legislatures to ensure teen access to DXM is restricted.

### Abuse mitigation plan results

The CHPA abuse mitigation campaign has focused on addressing the levers that influence a teen’s decision, including changing teen perceptions and attitudes toward abuse behavior; raising awareness among parents, caregivers, and influencers; and limiting teen access to these medicines. The campaign has also collected data monitoring trends in each campaign element, as well as measuring the overall annual prevalence of abuse.

#### Prevalence of abuse

Since 1975, the annual Monitoring the Future (MTF) survey, conducted by the University of Michigan with funding from the National Institutes of Health (NIH), has surveyed 40,000 to 50,000 students from the 8^th^, 10^th^, and 12^th^ grades in about 400 secondary schools throughout the U.S. In 2006, the MTF survey added a specific question on OTC cough medicine abuse; the reported annual prevalence rates are shown in Table 1. Since the 2010 FDA Advisory Committee meeting and the subsequent start of the CHPA abuse mitigation campaign, reports of abuse have decreased by approximately 35 % [15].

**Table 1:** Prevalence of Abuse: Monitoring The Future

	2006	2007	2008	2009	2010	2011	2012	2013	2014	2015
8^th^ Grade	4.2 %	4.0 %	3.6 %	3.8 %	3.2 %	2.7 %	3.0 %	2.9 %	2.0 %	1.6 %
10^th^ Grade	5.3 %	5.4 %	5.3 %	6.0 %	5.1 %	5.5 %	4.7 %	4.3 %	3.7 %	3.3 %
12^th^ Grade	6.9 %	5.8 %	5.5 %	5.9 %	6.6 %	5.3 %	5.6 %	5.0 %	4.1 %	4.6 %
Average	5.4 %	5.0 %	4.7 %	5.2 %	4.8 %	4.4 %	4.4 %	4.0 %	3.2 %	3.1 %

#### Teen perceptions and attitudes

Data on teen perceptions of risk and social disapproval associated with cough medicine abuse were monitored using data from the annual Partnership Attitude Tracking Survey (PATS) and from a CHPA-sponsored Online Tracking Study conducted by Hall & Partners. PATS, which surveyed teens in grades 9–12, assessed both their perception of risk and social disapproval [21]. The data (Table 2) shows that while there was little change in perception of DXM abuse as a risky behavior, there was a general disapproval of other teens using cough medicines to get high among the surveyed teenagers since 2011.

**Table 2:** Teen Attitude/Behavioral Changes: PATS Results

PATS (asked of teens)	2008	2009	2010	2011	2012	2013
Taking non-prescription cough or cold medicine to get high is risky (agree strongly/somewhat)	85 %	80 %	78 %	80 %	79 %	75 %
Taking non-prescription cough/cold medicine to get high has risks, including nausea and vomiting (agree strongly/somewhat)	N/A	N/A	N/A	88 %	88 %	86 %
Disapprove of others using non-prescription cough/cold products to get high (strongly disapprove/disapprove)	N/A	N/A	N/A	81 %	86 %	90 %
Taking non-prescription cough/cold medicine to get high is something cool kids do (agree strongly/somewhat)	N/A	N/A	N/A	29 %	36 %	26 %

The Hall & Partners Online Tracking Study [21] surveyed 14- to 20-year-olds before and after exposure to Internet content placed by the CHPA campaign. The data (Table 3) strongly reveal after forced exposure to campaign materials versus a baseline control group teens are much more likely to understand the social and physical risks, including thinking the behavior is socially unacceptable and would cause nausea and vomiting.

**Table 3:** Teen Attitude/Behavioral Changes: Hall & Partners Online Tracking Study

Hall & Partners Online Tracking Study (asked of teens)	Benchmark (2013)	After forced exposure (2015)	Difference in % points between forced exposure and benchmark
Please indicate how much you agree with the following statements about using non-prescription cough/cold medicine to get high			
Causes nausea and can lead to vomiting	63 %	92 %	+29
Don’t want to be seen as the kind of person who would do this	67 %	78 %	+11
Will make me look bad in front of friends	54 %	72 %	+18
Would you say the using non-prescription cough/cold medicine to get high is			
Feel physically sick	38 %	62 %	+24
Ashamed of	18 %	31 %	+13
Socially unacceptable	12 %	35 %	+23
Turns off friends	13 %	36 %	+23

#### Parental awareness and involvement

Data on parents’ awareness of and involvement in this issue come from two sources: 1) a CHPA-sponsored online survey conducted by David Binder Research in 2010, 2012, and 2013, and 2015 [21], and 2) the annual PATS survey [21]. As seen in Table 4, these data show nominal increases in parental monitoring and safeguarding of cough medicine in their household. However, results from both studies indicate that only about half of the surveyed parents are talking about cough medicine abuse.

**Table 4:** Parental Awareness/Involvement: David Binder Research Results

David Binder Research (asked of adults)	2010	2011	2012	2013	2015
Q. Which, if any, of the following actions have you taken?					
Monitored the amount of cough medicine in your household	31 %	N/A	39 %	37 %	40 %
Take steps to safeguard safe storage	30 %	N/A	38 %	33 %	44 %
Talked to your child about the dangers of cough medicine abuse	42 %	N/A	46 %	47 %	45 %
PATS (asked of adults)					
Had a conversation with teenager about using non-prescription cold or cough medicine to get high	58 %	57 %	67 %	59 %	N/A

#### Access

While access to DXM-containing medicines can be limited by increased parental monitoring, legislative and voluntary efforts to restrict access to those under 18 years of age continue. As of March 2016, ten states (California, New York, Virginia, Washington, Arizona, Louisiana, Kentucky, Tennessee, New Jersey, and Florida) have enacted age-restriction laws that prevent consumers under the age of 18 from purchasing products containing DXM. Although bipartisan, bicameral legislation was introduced in past Congresses, it has not yet passed. CHPA continues to lobby for federal and additional state legislation. Voluntary efforts by more than 20 national and regional retailers have also stopped selling to consumers under 18. According to the market research firm IRI between legislative and voluntary efforts, 88–90 % of the market does not sell dextromethorphan to those under 18.

There are limitations to the success of CHPA’s plan in that the breadth of information on how to abuse OTC cough medicine available on the Internet cannot be controlled. As long as there is information available, including dosage calculators and demonstration videos available through Google, Yahoo!, YouTube, Facebook, and elsewhere online, teens will always have the ability to learn about the behavior.

### Adverse event reporting system analysis

Another measure of the potential for abuse of DXM is adverse events reported to the FDA Adverse Event (AE) Reporting System database (FAERS). It is important to note that these events have been reported in association with DXM and do not prove that the drug was the causative agent. To assess whether there were changes in abuse-related serious adverse events associated with DXM over the first 3 years of CHPA’s abuse mitigation plan, CHPA commissioned a search of the FAERS database (2010–2013) using terms related to abuse. The search revealed a total of 56 adverse events in which DXM was listed as a primary suspect drug, including 32 with an associated outcome of death. Twenty-seven of these 32 reports included co-suspect drugs, including opiates and stimulants, and 19 were from the “2011 Annual Report of the American Association of Poison Control Centers’ National Poison Data System (NPDS): 29^th^ Annual Report.” Apart from an abrupt increase in AEs in the first quarter of 2013, which may have been driven by the publication of the 2011 NPDS annual report, this pattern was not sustained over the remaining quarters of 2013.

## Conclusions

The annual prevalence of OTC cough medicine abuse has been decreasing since 2006 when the question first appeared in Monitoring The Future and 3 years into CHPA’s preliminary education efforts. Similarly, the prevalence of abuse of a number of other substances has gone down over the past 9 years. We note, however, that since CHPA’s plan was presented at the 2010 FDA Advisory Committee meeting, the rate of decrease, according to MTF, appears accelerated. While a true cause-and-effect relationship cannot be assured, the authors, CHPA, and its member companies believe that the increased awareness of the issue since the 2010 FDA Advisory Committee meeting and the subsequent implementation of a well-delivered and targeted abuse mitigation plan that addressed the levers influencing teen decisions may be contributing to the observed reduction in abuse. Further support for this conclusion is found in a review of National Poison Data System intentional DXM abuse cases from 2000 to 2010, where Wilson et al. concluded it was likely a combination of legislative and educational efforts including CHPA’s efforts prior to our announcement of formal abuse mitigation plan goals and more comprehensive interventions that were responsible for what by 2010 was an observed plateau in DXM cases [22]. CHPA and the authors also believes that to continue providing cough symptom relief to those who require it, and because the results of targeted abuse mitigation efforts seem positive, this ingredient should remain accessible to consumers over the counter. Finally, CHPA hopes this case study on reducing substance abuse through targeted interventions can facilitate further discussion and provide learnings on effective approaches to preventing substance abuse, teen or otherwise.

## Abbreviations

AE, adverse event; CHPA, Consumer Healthcare Products Association; DEA, Drug Enforcement Administration; DXM, dextromethorphan; FAERS, Food and Drug Administration Adverse Event Reporting System; FDA, Food and Drug Administration; MTF, Monitoring the Future; NIDA, National Institute of Drug Abuse; NIH, National Institutes of Health; NMDA, N-methyl-D-aspartate; NPDS, National Poison Data System; OTC, over-the-counter; PATS, Partnership Attitude Tracking Survey; SAMSHA, Substance Abuse and Mental Health Service Administration; The Partnership, Partnership for Drug-Free Kids.
